# Dual Targeted Therapy with p53 siRNA and Epigallocatechingallate in a Triple Negative Breast Cancer Cell Model

**DOI:** 10.1371/journal.pone.0120936

**Published:** 2015-04-07

**Authors:** Cornelia Braicu, Valentina Pileczki, Laura Pop, Roxana Cojocneanu Petric, Sergiu Chira, Eve Pointiere, Patriciu Achimas-Cadariu, Ioana Berindan-Neagoe

**Affiliations:** 1 Research Center for Functional Genomics, Biomedicine and Translational Medicine, “Iuliu Hatieganu” University of Medicine and Pharmacy, Cluj-Napoca, Romania; 2 Faculty of Pharmacy, “Iuliu Hatieganu” University of Medicine and Pharmacy, Cluj-Napoca, Romania; 3 Faculty of Biology and Geology, Babeş-Bolyai University, Cluj-Napoca, Romania; 4 Haute École Louvain en Hainaut, Fleurus, Belgique; 5 Department of Surgery, The Oncology Institute " Prof Dr. Ion Chiricuta", Cluj-Napoca, Romania; 6 Department of Surgical Oncology and Gynaecological Oncology, “Iuliu Hatieganu” University of Medicine and Pharmacy, Cluj-Napoca, Romania; 7 Department of Immunology, “Iuliu Hatieganu” University of Medicine and Pharmacy, Cluj-Napoca, Romania; 8 Department of Functional Genomics and Experimental Pathology, The Oncology Institute " Prof Dr. Ion Chiricuta", Cluj-Napoca, Romania; 9 Department of Experimental Therapeutics M.D. Anderson Cancer Center Houston, Texas, United States of America; Virginia Commonwealth University, UNITED STATES

## Abstract

Triple-negative breast cancer (TNBC) is a highly aggressive phenotype that is resistant to standard therapy. Thus, the development of alternative therapeutic strategies for TNBC is essential. The purpose of our *in vitro* study was to evaluate the impact of p53 gene silencing in conjunction with the administration of a natural compound, epigallocatechingallate (EGCG). RT^2^Profiler PCR Array technology was used to evaluate the impact of dual treatment on the main genes involved in apoptosis in the Hs578T cell culture model of TNBC. Gene expression analysis revealed 28 genes were significantly altered (16 upregulated and 12 downregulated) in response to combined p53 siRNA and EGCG treatment. Further analysis revealed that p53 siRNA and EGCG dual therapy leads to the activation of pro-apoptotic genes and the inhibition of pro-survival genes, autophagy, and cell network formation. These results indicate that this dual therapy targets both the apoptotic and angiogenic pathways, which may improve treatment effectiveness for tumors resistant to conventional treatment.

## Introduction

Of all cancers, breast cancer has the highest incidence and mortality rate in Europe according to data from 2012 [1]. Approximately 15–20% of breast cancer cases are diagnosed as triple-negative breast cancer (TNBC), a highly aggressive clinical phenotype characterized by a lack of human epidermal growth factor receptor-2 (HER-2) overexpression, as well as a lack of estrogen and progesterone receptor expression [2, 3]. The overall survival rate of TNBC is less than 30% at five years after diagnosis due to its unique histological and molecular features, as well as the ineffectiveness of treatments and adjuvant hormone therapies [4]. TNBC represents a hostile histological subtype of breast cancer with limited medication options; therefore the development of alternative targeted therapies is important to improve the overall survival rates of TNBC patients [5].

The p53 gene, i.e. the ‘rebel angel’ according to Walerych [6], is the most frequently mutated gene in the pathology of breast cancer tumors [7]. Mutant p53 has an oncogenic role in tumorigenesis and metastasis [6]. The p53 protein is overexpressed in TNBC and is involved in the cellular stress response, repair and survival of damaged cells, and cell cycle arrest, [8], as well as resistance to apoptosis and inhibition of autophagy [6, 9, 10]. Increasing evidence shows that the p53 mutation is related to the activation of invasion and metastasis, as well as to inhibition of angiogenesis [6, 11], suggesting therapies involving p53 siRNA may target multiple molecular mechanisms as well as apoptosis [12].

Epigallocatechingallate (EGCG) is the most abundant compound found in green tea, and many research studies, in the last decade, have focused on its biological activities and mechanisms of action in cancer. EGCG inhibits several critical proteins that are involved in cancer cell progression [13], migration [14], and induction of apoptosis through the production of reactive oxygen species, induction of cell cycle progression, and inhibition of the NF-κB cell-signaling pathway [15].

To identify the physiological responsiveness of EGCG in tumor breast cancer cells, elucidating the molecular mechanisms and the molecular targets that trigger or inhibit a specific signaling pathway is essential [16]. In the present study, we investigated the response of *in vitro* breast cancer cells to multiple therapeutic targets by silencing mutant p53 through RNA interference mechanisms and investigating the inhibitory effect of EGCG on tumor cell survival, growth, and migration, and thereby the mechanism of treatment resistance using dual targeted therapy.

## Materials and Methods

### Cell culture

We purchased the TNBC cell line Hs578T, which expresses a mutant p53 gene, from the American Type Culture Collection for all experiments. Cells were maintained in high-glucose DMEM containing 10% fetal bovine serum, 2 mM L-glutamine, and 2 mM penicillin-streptomycin (all from Sigma-Aldrich, Germany) supplemented with 0.1% insulin. Cells were incubated in 5% CO_2_ incubator at 37°C.

### siRNA transfection

For mRNA analysis, cells were plated in 6-well plates at a seeding density of 5 × 10^5^ cells and simultaneously transfected, alone or in combination with 40 nmol p53-siRNA (Ambion, TX, USA) and EGCG (Sigma-Aldrich, St. Louis, MO, USA). The siPORT NeoFX Transfection Agent (Invitrogenby Life Technologies) being used for siRNA delivery, and cells were cultured in Opti-MEM I (Gibco-Invitrogen, Paisley, UK) reduced serum medium. Cells were harvested in TriReagent (Sigma-Aldrich, St. Louis, MO, USA) 24 hours after transfection and prepared for total RNA extraction. For autophagy and angiogenesis assays, we used 96-well plates and reduced the reagent volumes by one-tenth. All experiments being performed in triplicate.

### RNA extraction, qRT-PCR array and data analysis

Total RNA was isolated using the RNeasy Mini Kit (Qiagen, Hilden, Germany) and reverse transcribed to cDNA using RT2 First Strand Kit protocol according to the manufacturer’s instructions. A total of 102 μL cDNA were used for each Human Apoptosis RT2Profiler PCR Array plate. A reaction volume of 25 μL/well of RT2 SYBR Green Master mix with the appropriate RT2 Profiler Pathway “Signature” PCR Array was used according to manufacturer’s instructions. Gene expression assessment was done using Apoptosis PCR Array (PAHS-012Z), based on a standard protocol from Qiagen.

Gene expression was analyzed from three different experiments based on the ΔΔC_t_ method using PCR array data analysis software from SABioscience [17] (http://www.sabiosciences.com/pcrarraydataanalysis.php). Genes with a fold change ≤-1.5 or ≥1.5 were considered to be genes of interest. The Ingenuity System Pathway Analysis program was used to interpret and integrate the experimental data into biological networks.

### *In vitro* angiogenesis assay

We used the *In Vitro* Angiogenesis Assay Kit from Cayman Chemical (Cayman, USA) according to the manufacturer’s instructions with the following modifications. Plates were coated with 35 μl Cell-Based Extracellular Matrix Gel and incubated for 2 hours at 37°C to allow the gel to solidify before cell seeding. The cell network formation was evaluated under an inverted fluorescent microscope with filter for excitation at 485 nm and emission at 535 nm.

### Autophagy/cytotoxicity evaluation

We used the Autophagy/Cytotoxicity Dual Staining Kit (Cayman Europe, Estonia) according to the manufacturer’s protocol for both fluorescence microscopy and plate reader fluorescence detection. Plates were analyzed using a fluorescent microscope and a plate reader at excitation/emission wavelengths of 540/570 nm for propidium iodide and 350/520 nm for monodansylcadaverine detection, respectively.

## Results

### Gene expression data analysis

**Fig 1:**
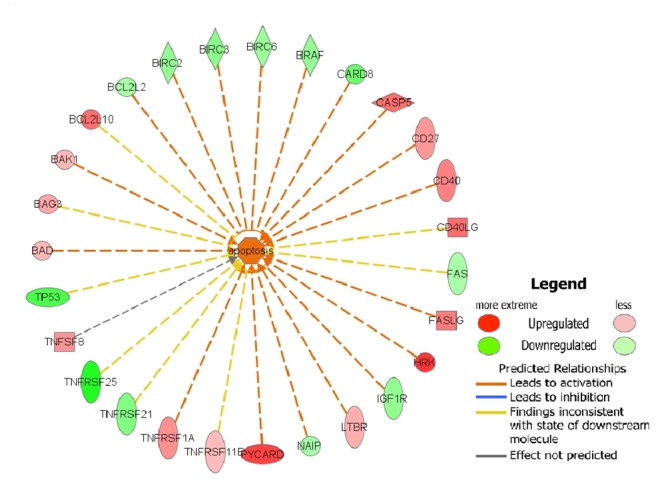
Gene network. Ingenuity analysis for the genes that stimulated/inhibited apoptosis in Hs578T cell line.

**Fig 2:**
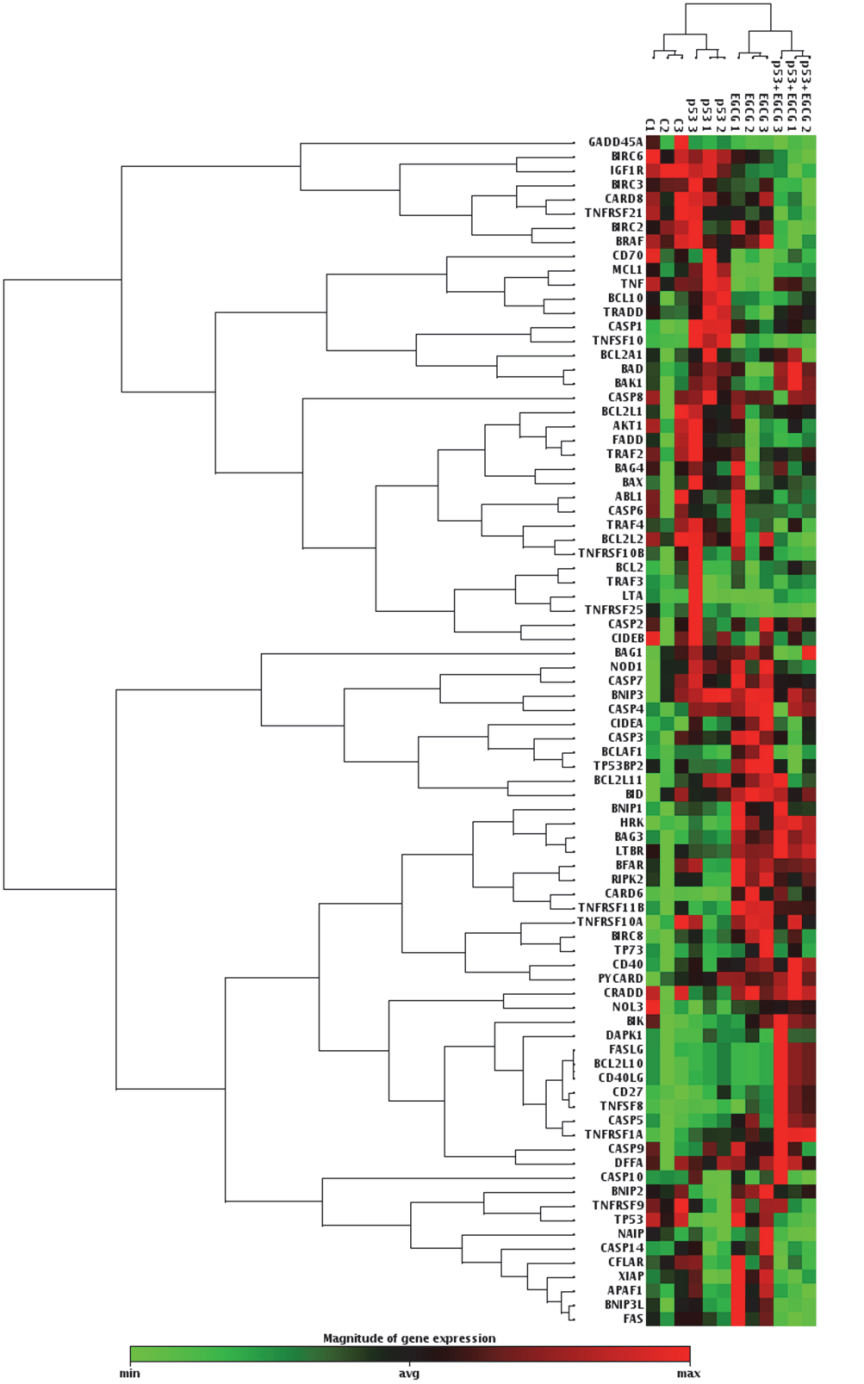
Expression matrix of triple negative breast cancer cells after treatment with p53 siRNA, EGCG, and combined treatment. According to the bar shown below the matrix, upregulated genes are represented in red and downregulated genes are represented in green. Small black squares represent unaltered genes. Each row represents color codes for a specific gene and each column indicates the treatment strategy, which was performed in triplicate.

Using qRT-PCR array technology, we examined the transcript levels of 84 genes involved in apoptosis from the Hs578T-cell line transfected with p53 siRNA and incubated with EGCG. Relative quantification of the transcripts using the 2^-ΔΔCT^method revealed 16 genes that were upregulated and 12 genes were downregulated in response to the combined treatment of p53 siRNA cells with EGCG (Table 1 and Figs 1 and 2).

**Table 1:** Genes that were found to be statistically significant in the experiment, classified by their fold regulation and stimulation/inhibition of genes involved in apoptosis pathway.

Gene	**Gene Symbol**	**Fold Regulation**
Harakiri, BCL2 interacting protein (contains only BH3 domain)	HRK	4.7327 ***
PYD and CARD domain containing	PYCARD	4.3853 *
CD40 ligand	CD40LG	3.3932 **
BCL2-like 10	BCL2L10	3.3932 **
Caspase 5, apoptosis-related cysteine peptidase	CASP5	3.3932 **
Fas ligand (TNF superfamily, member 6)	FASLG	3.0723 **
CD40 molecule, TNF receptor superfamily member 5	CD40	2.9201 *
Tumor necrosis factor receptor superfamily, member 1A	TNFRSF1A	2.5597 ***
Tumor necrosis factor (ligand) superfamily, member 8	TNFSF8	2.4612 **
CD27 molecule	CD27	2.3994 **
Caspase recruitment domain family, member 6	CARD6	2.1525 **
BCL2-associated athanogene 3	BAG3	2.0411 **
Lymphotoxin beta receptor (TNFR superfamily)	LTBR	1.8782 **
BCL2-associated agonist of cell death	BAD	1.6464 *
BCL2-antagonist/killer 1	BAK1	1.6388 *
Tumor necrosis factor receptor superfamily, member 11b	TNFRSF11B	1.5867 *
Fas (TNF receptor superfamily, member 6)	FAS	-1.5627 *
NLR family, apoptosis inhibitory protein	NAIP	-1.6253 *
BCL2-like 2	BCL2L2	-1.6029 *
Baculoviral IAP repeat containing 6	BIRC6	-1.8117 *
v-raf murine sarcoma viral oncogene homolog B1	BRAF	-1.9062 ***
Baculoviral IAP repeat containing 2	BIRC2	-1.9239 ***
Insulin-like growth factor 1 receptor	IGF1R	-1.9734 ***
Baculoviral IAP repeat containing 3	BIRC3	-2.1053 ***
Tumor necrosis factor receptor superfamily, member 21	TNFRSF21	-2.2408 *
Caspase recruitment domain family, member 8	CARD8	-2.6647 *
Tumor protein p53	TP53	-3.2058 ***
Tumor necrosis factor receptor superfamily, member 25	TNFRSF25	-3.965 *

*P-value <0.01

**P-value<0.001

***P-value<0.0001

Upon further analysis, we showed that the increases in gene expression occurred primarily in the Bcl-2 and tumor necrosis factor receptor superfamilies, which are associated with activation of apoptotic mechanisms (Table 1 and Fig 1). By contrast, the BIRC family members, which inhibit apoptosis by preventing the proteolysis of procaspase-3, procaspase-6, and procaspase-7, were downregulated.

### Angiogenesis and autophagy/cytotoxicity evaluation

**Fig 3:**
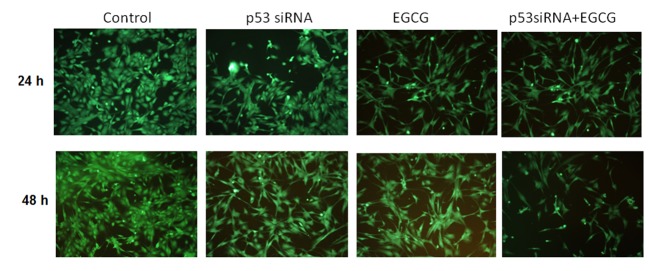
Cell network formation. Microscopical evaluation was done in the presence of the selected treatment scenarios at 24 and 48 h post-treatment in Hs578T cell line.

**Fig 4:**
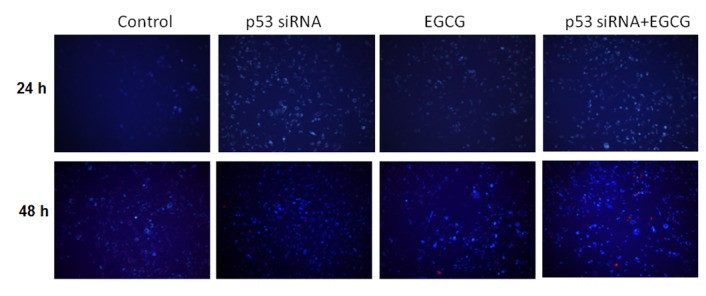
Autophagy evaluation. Microscopical evaluation was done in the presence of the selected treatment scenarios at 24 and 48 h post-treatment in Hs578T cell line.

Figs 3 and 4 show the effect of p53 gene knockdown and combined treatment with EGCG in blocking angiogenesis and autophagy. Hs578T cell network formation was significantly reduced in the cells treated with EGCG for 24 and 48 h, respectively, compared with control-treated cells.

## Discussion

Most studies of TNBC are focused on the identification of novel chemotherapeutics targeting pathways involved in angiogenesis, growth, survival, or activation of apoptosis [3–5, 6]. Because TNBC has a highly heterogeneous pathology, most studies address the idea of combined therapy. The purpose of our study was to investigate whether combined treatment with p53 siRNA and EGCG increased apoptosis in the TNBC cancer cell line, Hs578T. Using PCR array approach, we showed that EGCG altered gene expression and promote apoptosis, decrease cell survival, and reduce angiogenesis and autophagy in cells with p53 siRNA. These findings may provide insight on genetic-based approaches for treating TNBC based on the specific activation of pro-apoptotic genes and inhibition of pro-survival genes in response to combined treatment with p53 siRNA and EGCG. Our findings suggest that the combined p53 siRNA and EGCG treatment increased apoptosis more than either of these treatments alone, which may contribute to increased TNBC cell death.

Increasing evidence has shown that expression of the p53 gene is related to breast cancer prognosis [3, 6, 18]. A previous study on HeLa cells shows that cancer therapy using p53 siRNA specifically triggers apoptotic mechanisms [19, 20, 21], and increases the efficiency of other therapeutic agents by increasing the sensitivity of cancer cells to apoptosis [20].

The p53 protein is an important mediator of various cellular processes, such as modulation of senescence, apoptosis, and cell cycle genes [6, 7]. Recent clinical trials have demonstrated the role of p53 siRNA and the efficacy of RNA interference-based drugs in general in anticancer therapy [22, 23], although the precise transcriptional mechanisms by which p53 siRNA initiates and supports apoptosis still need to be clarified. The extrinsic signaling pathway of apoptosis is based on the activation of so-called ‘death receptors,’ whereas the intrinsic mechanism is activated by modifications in DNA structure. After the mitochondrial membrane is depolarized, cytochrome c is released into the cytoplasm from the intermembrane space of the mitochondria. The result of both extrinsic and intrinsic pathways is the activation of caspases and ultimately cell death [6, 7]. p53 increase the recovery of cells damaged by therapy, thus acting as a survival factor to prevent mitotic catastrophe and to provide a basis for dual therapies [24].

One possible mechanism by which the two apoptotic pathways converge is based on the synergistic effect of p53 together with the administration of EGCG or other therapeutic compounds. Similar to our previous findings [25], combined p53 siRNA and EGCG treatment minimized the activation of the anti-apoptotic genes (such as BAG3, XIAP, and RIPK2) related to treatment resistance, which may further increase cancer cell sensitivity to treatment.

Along with the present study, recent experimental studies have shown that inhibition of autophagy or Fas signaling may be novel therapeutic targets for TNBC therapy [26]. In addition, inhibition of autophagy increased the therapeutic response in anthracycline-sensitive and-resistant TNBC, emphasizing the importance of this mechanism in drug resistance [27]. The inhibition of autophagy shifts the expression of the p53 protein, Bcl-2 family proteins, and the ratio of Bax/Bcl-xL proteins, which promotes apoptosis in MDA-MB-231 cells (a TNBC model similar to Hs578T) [28].

Our previous studies show that EGCG suppresses migration and invasion of TNBC cells [29]. We confirmed this in the present study with the evaluation of the cell network formation and the activation of drug resistance genes [25]. Therefore, the combination of p53 siRNA and EGCG may also increase efficacy of treatment by inhibiting cell network formation and activating autophagy.

Multi-targeted therapy optimizes efficacy of anti-tumor treatment. These data may be particularly useful in TNBC, a form of breast cancer that is highly resistant to current cancer therapies and has an average survival rate of less than three years. Therefore, our proposed treatment is particularly advantageous because it specifically targets anti-apoptotic, anti-angiogenic, and anti-autophagic mechanisms of these cancer cells.

## Conclusion

Comprehension of how a particular therapeutic combination affects every tumor compartment paves the way for the discovery of novel drugs or therapeutic strategies. Our preliminary results showed that combining EGCG with p53 siRNA enhanced the antitumoral effect on the TNBC cancer cell line, by specifically activation of apoptosis and autophagy.

This study provides novel insight on new multifaceted breast cancer therapies that target a genetic component, such as the overexpression of mutant p53, and administer in conjunction with a natural compounds such as EGCG.

## Supporting Information

S1 Data Set PCR-array raw data. (XLS)
